# Editorial: Recent Developments in Neuroanatomical Terminology

**DOI:** 10.3389/fnana.2019.00080

**Published:** 2019-08-07

**Authors:** Hans J. ten Donkelaar, Luis Puelles

**Affiliations:** ^1^Department of Neurology, Radboud University Medical Center, Nijmegen, Netherlands; ^2^Department of Human Anatomy and Psychobiology, Facluty of Medicine, Universidad de Murcia, Murcia, Spain

**Keywords:** terminology, prosomeric model, cerebral cortex, thalamus, neural progenitor cells, neuron types, tracts

A recent revision of the terminology of the sections titled the “Central nervous system” (CNS) and the “Peripheral nervous system” (PNS) within the *Terminologia Anatomica* (1998) and the *Terminologia Histologica* (2008) has been posted to the open part of the Federative International Programme for Anatomical Terminology (FIPAT) website (http://FIPAT.library.dal.ca) as the official FIPAT terminology for the nervous system, the *Terminologia Neuroanatomica* (TNA, 2017). A third chapter deals with the sensory organs. The major differences between the TNA and the TA and TH have been outlined in an introductory paper (ten Donkelaar et al., 2017). For an illustrated version of the TNA, see ten Donkelaar et al. (2018).

In general, the TNA uses a more natural hierarchical and embryologically-based classification of brain structures for the prosencephalon (forebrain), following the prosomeric model (Puelles, 2013; Puelles et al., 2013). Neuron types are implemented for all of the sections. Given these novelties, involving a framework change in the prevalent neuromorphological descriptive paradigm (that is, the current prosomeric model vs. Herrick's columnar model), and their potential impact on the future communication of neuroanatomical research results, the scientific community might profit from a wider discussion of the FIPAT's decisions. Accordingly, discussion focused on the following topics:

- **Further development of a developmental ontology**. Three papers discuss the further implementation of a developmental ontology into neuroanatomical terminology: (1) The subdivision of the forebrain based on embryological and genoarchitectonic studies; the forebrain is subdivided into the caudal prosencephalon, giving rise to the midbrain-diencephalon (midbrain, pretectum, thalamus with epithalamus, prethalamus, and related tegmental parts), and the rostral prosencephalon, giving rise to the hypothalamus, the eyes, and the entire telencephalon. Puelles' review surveys midbrain, diencephalic, and hypothalamic neuroanatomical concepts and various recent findings whose prosomeric pregnancy conflicts with columnar tradition, leaving a complex scenario with many terminological problems to be gradually resolved within the field. He also contributes an updated prosomeric concept of the diencephalic-telencephalic transition. (2) New definition of midbrain boundaries and corresponding alar subdivisions; the transgenic approach establishes a new concept of the isthmocerebellar or prepontine hindbrain (Watson et al., 2017), conventionally misidentified as a part of the midbrain. Another novel aspect touches the conventional pons, which is subdivided into prepontine, pontine, and retropontine or pontomedullary hindbrain neuromeric domains, restricting the term pons to the basilar part of the pons. The contribution by Watson et al. recommends a new brain stem nomenclature based on developmental gene expression, progeny analysis, and fate mapping. (3) In the TNA, a modernized version of the blood vessels of the brain with clinical subdivisions is included to ensure it contains a more or less complete list of terms for the human nervous system. The paper by Ferran's group attempts a prosomeric molecular-marker analysis of the early vascularization of the embryonic mouse forebrain and presents a tentative topological map relating human brain vessels to specifc segmental and dorsoventral units, also touching on some terminological issues (Puelles et al.).
- **Common terminology for cerebral cortex and thalamus**. Three papers deal with aspects of the nomenclature for the cerebral cortex and the thalamus: (1) one aiming for a common terminology for the gyri and sulci of the cerebral cortex (ten Donkelaar et al.); (2) a second on the cytoarchitectonic areas of the gyrus ambiens (Insausti et al.), incorporating the Brodmann area 34 into the entorhinal cortex; and (3) a third on subdivisions for the thalamic nuclei. Mai and Majtanik contributed an extensive review of the various terminologies used for thalamic nuclei, using a new volumetric approach to characterize the significant subdivisions, normalizing the individual thalamus shapes in MNI space, which allows comparison of the nuclear regions delineated by the different authors. Their final scheme of the spatial organization provided the frame for the selected terms for the subdivisions of the human thalamus using on the (modified) terminology of the TNA.
- **White matter tracts**. Two papers deal with white matter tracts, which in the TNA follows the Swanson and Bota (2010) classification as central roots, intrinsic tracts, commissural connections and long tracts, divided into ascending and descending tracts: (1) Baud et al. address a new scheme for the representation of white matter in the CNS. In this approach, white matter is directly attached to the CNS, and no longer considered part of the brain segments. The new classification of white matter tracts selects the origin as the primary criterion and the type of tract as the secondary criterion. It follows a top-down approach from telencephalon to spinal cord; (2) Mandonnet et al. discuss the nomenclature of the human white matter association pathways and propose a new nomenclature based on the structural wiring diagram of the human brain; and (3) in a Commentary, Panesar and Fernández-Miranda emphasize that cortical connectivity should be identified on the basis of their origin, termination and axonal properties.
- **Neuron types**. In the TNA, the terms for the various types of neurons provided by Bota and Swanson (2007) are used. Three papers deal with aspects of this topic: (1) one on auditory nomenclature, combining name recognition with anatomical description, which should help future generations in learning the structure-function correlates of the inner ear more easily (Fritzsch and Elliott); (2) a second on neural progenitor cell (NPC) nomenclature, including embryonic and adult precursor cells of the cerebral cortex and the hippocampus, increasing our knowledge of what is ultimately most important, i.e., understanding NPC function in the developing as well as in the adult CNS (Martínez-Cerdeño and Noctor); and (3) a major one on neuron names in a gene- and property-based format, with special reference to cortical neurons (Shepherd et al.). Precision in neuron name is increasingly needed now that we are entering a new era in which classic anatomical criteria are only the beginning of defining the identity of a neuron. New criteria include patterns of gene expression, membrane properties, neurotransmitters and neuropeptides, and physiological properties. Related to this topic is (4) a paper on navigating the murine brain aimed toward best practices for determining and documenting neuroanatomical locations in experimental studies (Bjerke et al.).

The suggestions made to improve the TNA will be considered in the next version of the TNA. Neuroanatomical terminology remains an actively ongoing endeavor.

## Author Contributions

HtD and LP designed the Research Topic, invited contributors and edited most of the manuscripts.

### Conflict of Interest Statement

The authors declare that the research was conducted in the absence of any commercial or financial relationships that could be construed as a potential conflict of interest.
